# National survey questionnaire on the diagnosis and treatment status of *Helicobacter pylori* in peptic ulcer bleeding disease

**DOI:** 10.3389/fpubh.2024.1433139

**Published:** 2024-09-11

**Authors:** Zongdan Jiang, Yaokun Ding, Chao Li, Zhenyu Zhang

**Affiliations:** ^1^Department of Gastroenterology, Nanjing First Hospital, Nanjing Medical University, Nanjing, Jiangsu, China; ^2^Department of Emergency, Nanjing First Hospital, Nanjing Medical University, Nanjing, Jiangsu, China

**Keywords:** peptic ulcer bleeding, *Helicobacter pylori*, questionnaire, consensus, different level hospitals

## Abstract

**Background and objective:**

The Maastricht VI/Florence Consensus and Chinese National Consensus Report provide comprehensive guidelines for treating *Helicobacter pylori* infection. This study aimed to assess physicians' understanding of and adherence to this consensus in different hospitals.

**Methods:**

Chinese medical staff attending gastrointestinal conferences across various regions were selected for this study. The questionnaire included: 1. the number of patients with peptic ulcer bleeding in hospitals of different levels annually and the diagnostic methods used for *H. pylori*; 2. whether routine *H. pylori* examination was conducted and the specific methods employed; and 3. Treatment plans for *H. pylori* eradication; 4. The mean follow-up duration after treatment 5. Plans for re-eradication in cases of *H. pylori* treatment failure.

**Results:**

Across all levels of Chinese hospitals, the urea breath test was the most commonly used method for detecting *H. pylori* infection. Most primary (81.53%), secondary (89.49%), and tertiary (91.42%) centers opted for a 14-day quadruple regimen. The preferred antibiotic regimen at all hospital levels was amoxicillin+clarithromycin, with rates of 63.69, 58.08, and 59.27% in the primary, secondary, and tertiary hospitals, respectively. The rates of *H. pylori* re-examination were 68.15, 87.07, and 87.46% in the primary, secondary, and tertiary hospitals. If *H. pylori* eradication failed, hospitals at different levels choose to replace the initial plan.

**Conclusion:**

There is a need for an enhanced understanding of and adherence to guidelines for *H. pylori* among physicians in hospitals at all levels.

## Introduction

Upper gastrointestinal bleeding refers to bleeding occurring in the gastrointestinal tract proximal to the ligament of Treitz. Failure to promptly treat upper gastrointestinal bleeding results in 2–10% of annual deaths globally (1). Peptic ulcer disease is a common cause of upper GI bleeding, accounting for 30–60% of cases (2). Peptic ulcers are ulcerative conditions that primarily affect the stomach and duodenum and typically present with symptoms including abdominal distension, belching, and nausea. Severe disease can lead to gastrointestinal bleeding and even malignancy (3). The incidence of peptic ulcers ranges from 6 to 15% in Europe and America and ~10–12% in China (4). The development of peptic ulcers is closely associated with factors such as glucocorticoid use, thinning of the gastrointestinal mucosa, genetic susceptibility, excessive gastric acid production, and infection with *H. pylori* (5–7). *H. pylori* infection significantly contributes to developing peptic ulcers because the toxins this bacterium produces trigger immune responses that lead to mucosal damage.

*H. pylori* infection affects approximately half of the global population, with an infection rate of about 46.7% among the Chinese population (8, 9). The current status of *H. pylori* diagnosis and treatment can be summarized as having “three highs and one low”: high infection rate, high drug resistance rate, high pathogenicity, and low eradication rate. Antibiotic resistance in *H. pylori* is the primary factor contributing to the decline in the eradication rate (10). Globally, *H. pylori* antibiotic resistance is increasing, particularly in China. The Maastricht VI/Florence consensus was published in Gut in 2022 to address this concern. Following this consensus, the *Helicobacter pylori* Group of the Gastroenterology Branch of the Chinese Medical Association released the sixth national consensus report on the management of *H. pylori* infection in 2022. However, whether physicians from different hospitals across the country are well-acquainted with the content and application of these guidelines remains uncertain. A questionnaire was distributed in June 2023 to investigate the current diagnosis and treatment practices for *H. pylori*-related peptic ulcer bleeding among physicians in hospitals of varying levels. This study aimed to assess physicians' understanding of the consensus guidelines, particularly among gastroenterologists from hospitals of different levels.

## Methods

### Study participants

The study participants were Chinese medical staff who attended digestion conferences held in various regions of the country from June 2023 to November 2023. Medical staff were categorized into primary, secondary, and tertiary hospitals. Primary hospitals are healthcare institutions that directly provide the community with comprehensive services, such as medical treatment, prevention, rehabilitation, and healthcare. Secondary hospitals are regional hospitals that provide medical and health services across several communities and are technical centers for regional medical prevention. Tertiary hospitals provide medical and health services across regions, provinces, cities, and nationwide. These are medical prevention technology centers with comprehensive medical, teaching, and research capabilities. This study was approved by the Medical Ethics Committee of the Nanjing First Hospital.

### Research methods

The research was conducted using a questionnaire distributed through a WeChat mini program, which allowed for convenient distribution of the questionnaire and data collection. The questionnaire included the following topics.

1. Annual number of patients with peptic ulcer bleeding in hospitals of different levels and *H. pylori* diagnostic methods employed.
2. Determining whether patients with peptic ulcer bleeding undergo routine *H. pylori* examination and the specific methods used for *H. pylori* testing.
3. Assessing the timing of *H. pylori* eradication, treatment plan and choice of antibiotics for *H. pylori* eradication.
4. Investigating the duration of follow-up after treatment.
5. Examination of the timing and plan for re-eradication in cases of *H. pylori* treatment failure.

### Statistical analysis

The collected data were analyzed using SPSS 22.0. Descriptive statistics were used to present count data as percentages. Intergroup comparisons were performed using the chi-square test or Fisher's exact probability method. Statistical significance was set at *p* < 0.05.

## Results

### Demographic characteristics of participants

**Figure 1:**
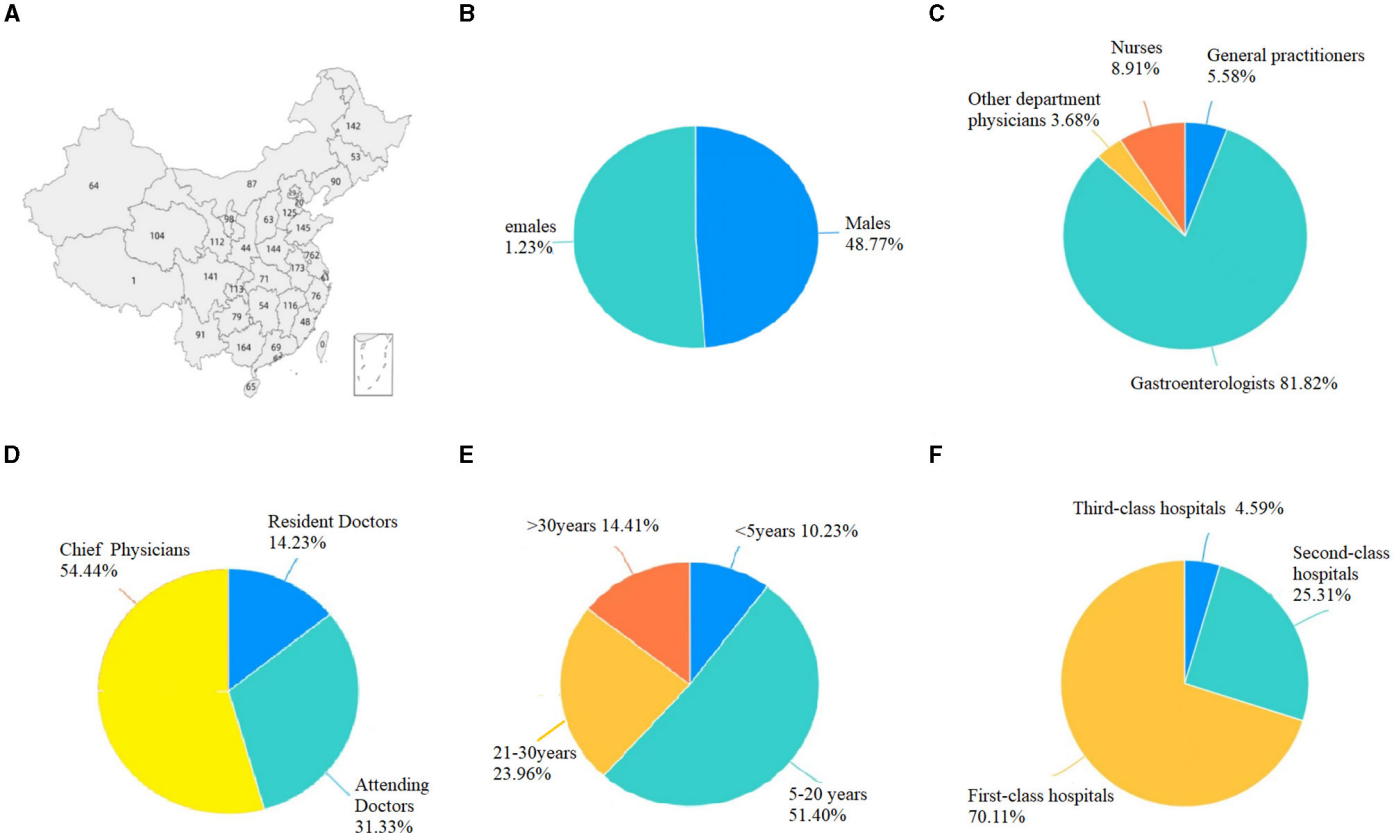
Demographic characteristics of participants. **(A)** The distribution of Chinese medical staff and the provinces where they worked. **(B)** Gender distribution of medical staff. **(C)** Composition of medical staff. **(D)** Levels of the surveyed physicians. **(E)** Years of service of the surveyed physicians. **(F)** The levels of the hospital where the surveyed physicians are located.

The WeChat mini program received 3,422 survey questionnaires, as depicted in Figure 1A, illustrating the distribution of Chinese medical staff and the provinces where they worked. Among the Chinese medical staff participating in the questionnaire survey, men accounted for 48.77% (1,669/3,422), and women accounted for 51.23% (1,753/3,422) (Figure 1B). Gastroenterologists, general practitioners, nurses, and other department physicians constituted 81.82% (2,800/3,422), 5.58% (191/3,422), 8.91% (305/3,422), and 3.68% (126/3,422) of the sample, respectively (Figure 1C). Among the Chinese medical staff, Chief Physicians, Attending Doctors, and Resident Doctors represented 54.44% (1,863/3,422), 31.33% (1,072/3,422), and 14.23% (487/3,422), respectively (Figure 1D). The results indicated that 10.23% (350/3,422) had ≤ 5 years' experience, 51.40% (1,759/3,422) had 520 years, 23.96% (820/3,422) had worked 21–30 years, and 14.41% (493/3,422) had been working ≥30 years (Figure 1E). Questionnaire respondents worked in primary hospitals, 4.59% (157/3,422), secondary hospitals, 25.31% (866/3,422), and tertiary hospitals, 70.11% (2,399/3,422) (Figure 1F).

### The annual number of patients with peptic ulcer bleeding in hospitals of different levels and the *H. pylori* diagnostic methods applied

Among primary hospitals, 127 had fewer than 50 individuals requiring hospitalization for peptic ulcer bleeding annually, 21 admit 50–100 patients annually, and nine admit over 100 patients annually. In secondary hospitals, 280 admitted ≤ 50 patients per year because of peptic ulcer bleeding, 351 admit 50–100 patients per year, and 235 admitted >100 patients annually. Among the tertiary hospitals, 172 admitted no more than 50 patients per year, 636 admit between 50–100 patients per year, and 1,591 admit more than 100 patients per year (Table 1).

**Table 1:** Annual number of patients with peptic ulcer bleeding in hospitals of different levels, and the *H. pylori* diagnostic methods applied.

**Characteristics**	**Primary hospitals**	**Secondary hospitals**	**Tertiary hospitals**	***p*-value**
**Annual number of patients hospitalized due to peptic ulcer bleeding**				
< 50	127	280	172	< 0.01
50–100	21	351	636	< 0.01
>100	9	235	1,591	< 0.01
***H. pylori*** **diagnostic methods**				
Rapid urease test	47	148	874	< 0.01
^13^C urea breath test	91	512	2,043	< 0.01
^14^C urea breath test	61	700	1,774	< 0.01
Serological antibody test	48	350	1,603	< 0.01
Fecal antigen test	10	54	417	< 0.01
Pathological tissue test	38	255	1,320	< 0.01
Culture-based drug sensitivity test	13	66	476	< 0.01
Fluorescence PCR	7	28	194	< 0.01
None	26	15	4	< 0.01

We found that 16.56% (26/157) of primary hospitals do not perform diagnostic testing for *H. pylori*, secondary hospitals accounted for 1.73% (15/866), and tertiary hospitals for 0.1% (4/2,399). The top five methods were the rapid urease test, ^13^C urea breath test, ^14^C urea breath test, the serological antibody test, and the pathological tissue test, with the urea breath test being the most commonly used (Table 1).

### *H. pylori* examination in hospitals of different levels

In primary hospitals, 68.79% (108/157) of the patients underwent *H. pylori* examination during hospitalization, 16.56% (26/157) underwent outpatient examination after discharge, and 14.65% (23/157) did not undergo examination. During hospitalization in secondary hospitals, 80.72% (698/866) of the patients underwent *H. pylori* examination, 17.09% (148/866) underwent outpatient examination after discharge, and 2.19% (19/866) did not undergo examination. In tertiary hospitals, 76.01% (1,824/2,399) of the patients underwent *H. pylori* examination during hospitalization, 22.45% (539/2,399) underwent outpatient examination after discharge, and 1.54% (37/2,399) did not undergo examination (Table 2).

**Table 2:** Routine *H. pylori* examination and examination methods for patients with peptic ulcers and bleeding in hospitals of different levels.

**Characteristics**	**Primary hospitals**	**Secondary hospitals**	**Tertiary hospitals**	***p*-value**
**Routine** ***H. pylori*** **examination**				
During hospitalization	108	698	1,824	0.01
After discharge	26	148	539	0.01
Did not undergo examination	23	19	37	0.00
**Methods**				
Rapid urease test	14	70	236	0.00
Urea breath test	84	618	1,542	0.00
Serological antibody test	15	67	313	0.31
Fecal antigen test	4	10	30	0.40
Pathological tissue test	13	48	164	0.28
Fluorescence PCR	1	2	8	0.59
None	26	51	108	0.00

The methods used for *H. pylori* examination in primary hospitals included rapid urease test (8.92%, 14/157), urea breath test (53.5%, 84/157), serological antibody test (9.55%, 15/157), fecal antigen test (2.55%, 4/157), histopathological test (8.28%, 13/157), and polymerase chain reaction (PCR) testing (0.64%, 1/157). In secondary hospitals, the methods included rapid urease test in 8.08% (70/866), urea breath test in 71.36% (618/866), serological antibody test in 7.74% (67/866), fecal antigen test in 1.15% (10/866), histopathological test in 5.54% (48/866), and PCR testing in 0.23% (2/866). In the tertiary hospitals, the methods used were as follows: rapid urease test, 9.83% (236/2,399); urea breath test, 64.22% (1,542/2,399); serological antibody test, 13.04% (313/2,399); fecal antigen test, 1.25% (30/2,399); histopathological test, 6.83% (164/2,399); and PCR test, 0.33% (8/2,399) (Table 2).

### Timing and regimen of *H. pylori* eradication

In primary hospitals, *H. pylori* eradication was achieved in 21.02% (33/157) of patients during hospitalization; 50.31% (79/157) were cured after discharge, 17.83% (28/157) were cured after ulcer healing, and 10.83% (17/157) did not achieve eradication. During hospitalization in secondary hospitals, 31.99% (277/866) of the patients underwent *H. pylori* eradication, 51.96% (450/866) underwent post-discharge diagnosis and eradication, 13.05% (113/866) underwent ulcer healing eradication, and 3% (26/866) underwent non-eradication. In tertiary hospitals, *H. pylori* eradication was achieved in 22.87% (548/2,399) of patients; 58.90% (1,413/2,399) were cured after discharge, 17.41% (418/2,399) were cured after ulcer healing, and 0.83% (20/2,399) did not achieve eradication (Table 3).

**Table 3:** Timing and regimen of *H. pylori* eradication and selection of antibiotics for patients with peptic ulcers and bleeding in hospitals of different levels.

**Characteristics**	**Primary hospitals**	**Secondary hospitals**	**Tertiary hospitals**	***p*-value**
**Timing of** ***H. pylori*** **eradication**				
During hospitalization	33	277	548	0.00
After discharge	79	450	1,413	0.07
After ulcer healing	28	113	418	0.01
Non eradication	17	26	20	0.00
**Regimen of** ***H. pylori*** **eradication (14 days)**				
High-dose dual therapy	11	27	82	0.05
Triple drug therapy	13	58	107	0.01
Quadruple regimen containing bismuth	128	774	2,194	0.00
Quadruple regimen non-containing bismuth	3	6	14	0.13
Other options	2	0	3	0.01
**Selection of antibiotic regimens**				
Amoxicillin + furazolidone	15	211	561	0.00
Amoxicillin + clarithromycin	100	503	1,422	0.42
Amoxicillin + levofloxacin	19	39	126	0.00
Tetracyclines + metronidazole	2	4	18	0.49
Tetracyclines + furazolidone	3	8	14	0.10
Amoxicillin + metronidazole	12	82	149	0.01
Amoxicillin + tetracyclines	3	17	92	0.02
Other options	3	2	17	0.04

Most primary, secondary, and tertiary hospitals selected a quadruple 14-day bismuth regimen, accounting for 81.53, 89.49, and 91.42%, respectively). The high-dose dual therapy 14-day regimen ranked third, accounting for 7.01, 3.12, and 3.42%, respectively (Table 3).

Amoxicillin and clarithromycin were the most commonly chosen options at all levels of hospitals, accounting for 63.69% (100/157) of primary hospitals, 58.08% (503/866) of secondary hospitals, and 59.27% (1,422/2,399) of tertiary hospitals. In primary hospitals, amoxicillin + levofloxacin accounted for 12.10% (19/157), amoxicillin + furazolidone 9.55% (15/157), and amoxicillin + metronidazole 7.64% (12/157). In secondary and tertiary hospitals, the follow-up plans included amoxicillin + furazolidone at 24.36 and 23.41% (211/866, 561/2,399), amoxicillin + metronidazole at 9.47 and 6.21% (82/866, 149/2,399), and amoxicillin + levofloxacin at 4.50 and 5.25% (39/866, 126/2,399), respectively (Table 3).

### Timing of re-examination for *H. pylori* in patients with peptic ulcers and bleeding after eradication

The results indicated that after the eradication of *H. pylori*, the rates of *H. pylori* re-examination after stopping medication for 2 weeks, 4 weeks, 6 months, and no re-examination after stopping medication in primary hospitals were 20.38% (32/157), 68.15% (107/157), 7.01% (11/157), and 4.46% (7/157), respectively. In the secondary hospitals, the rates were 8.78% (76/866), 87.07% (753/866), 3.35% (29/866), and 0.81% (7/866), respectively. Tertiary hospitals had rates of 8.54% (205/2,399), 87.46% (2,099/2,399), 3.79% (91/2,399), and 0.21% (5/2,399), respectively (Table 4).

**Table 4:** Time for re-examination of *H. pylori* in patients with peptic ulcers and bleeding in hospitals of different levels after eradication of *H. pylori*.

**Characteristics**	**Primary hospitals**	**Secondary hospitals**	**Tertiary hospitals**	***p*-value**
**Time of re-examination for** ***H. pylori***				
Stopping medication for 2 weeks	32	76	205	0.00
Stopping medication for 4 weeks	107	753	2,099	0.00
Stopping medication for 6 months	11	29	91	0.09
No re-examination	7	7	5	0.00

### The time and plan selection for re-eradication of *H. pylori* in patients with treatment failure

The re-eradication times for *H. pylori* after failed eradication were 27.39% (43/157), 31.85% (50/157), 36.94% (58/157), and 3.82% (6/157) in primary hospitals after 1, 3, 6, and 12 months, respectively. In secondary hospitals, the rates were 22.06% (191/866), 40.53% (351/866), 36.14% (313/866), and 1.27% (11/866). Tertiary hospitals had rates of 15.83% (380/2,399), 36.23% (869/2,399), 45.69% (1,096/2,399), and 2.25% (54/2,399), respectively (Table 5).

**Table 5:** The time and plan selection for re-eradication of *H. pylori* failed patients.

**Characteristics**	**Primary hospitals**	**Secondary hospitals**	**Tertiary hospitals**	***p*-value**
**Re-eradication time for** ***H. pylori*** **after failed eradication**				
After 1 month	43	191	380	0.00
After 3 months	50	351	869	0.03
After 6 months	58	313	1,096	0.00
After 12 months	6	11	54	0.06
**The plan for re-eradication after failed eradication of** ***H. pylori***				
Changing the plan	92	566	1,855	0.00
Extending the course of treatment	7	12	25	0.00
Changing the plan + extending the course of treatment	48	243	424	0.00
Adding traditional Chinese medicine	4	17	23	0.03
Adding probiotics	3	20	63	0.75
Other methods	3	7	10	0.04

The selection of a plan for re-eradication after the failed eradication of *H. pylori* included changing the plan, extending the course of treatment, changing the plan and extending the course of treatment, adding traditional Chinese medicine, adding probiotics, and other methods. Choices in primary hospitals were 58.60% (92/157), 4.46% (7/157), 30.57% (48/157), 2.25% (4/157), 1.91% (3/157), and 1.91% (3/157), respectively. Secondary hospitals had choices of 65.47% (566/866), 1.39% (12/866), 28.06% (243/866), 1.96% (17/866), 2.31% (20/866), and 0.81% (7/866), respectively. Tertiary hospitals showed choices of 77.30% (1,855/2,399), 1.04% (25/2,399), 17.66% (424/2,399), 0.96% (23/2,399), 2.62% (63/2,399), and 0.42% (10/2,399), respectively (Table 5).

## Discussion

Peptic ulcers are a prevalent disease worldwide, and their most significant complication is bleeding, which can be life-threatening in severe cases. Extensive domestic and international research has confirmed that *H. pylori* is the primary causative factor of peptic ulcers. The detection rate of *H. pylori* in gastric mucosal biopsy samples from peptic ulcer patients is reported to exceed 80% in gastric ulcer patients and 90–100% in duodenal ulcer patients. The risk of peptic ulcer development is significantly increased in individuals infected with *H. pylori*. Eradicating *H. pylori* has been shown to reduce the annual recurrence rate of gastric and duodenal ulcers to < 20%, whereas patients who have not undergone eradication treatment can experience an annual recurrence rate of >60% (11). Therefore, consensus guidelines recommend *H. pylori* eradication as an effective measure for preventing and treating peptic ulcers.

This survey included practitioners in hospitals in provinces country-wide except Taiwan. The participants consisted mainly of gastroenterologists, accounting for 81.82%, and 89.77% had more than 5 years of experience. Most (85.77%) held positions as professors or attending doctors, primarily working in tertiary hospitals. Therefore, this survey provided valuable insights into the awareness and application of Consensus Guidelines among doctors at different hospital levels.

The survey revealed that hospitals at different levels had patients admitted for peptic ulcer bleeding. As the hospital level increased, the number of patients also increased, potentially indicating a higher trust of patients in tertiary hospitals. Various methods have been employed to diagnose *H. pylori*, with the urea breath test being the most commonly used across hospital levels. Other frequently employed methods included serological antibody testing, histopathological testing, and rapid urease testing, fulfilling the basic requirements for diagnosing and re-examining *H. pylori* patients. The proportion of bacterial cultures and drug sensitivity testing exceeded that of fecal antigen testing. Monoclonal fecal antigen testing has similar accuracy to the urea breath test but has limitations regarding adult acceptance due to the need for stool collection. Monoclonal fecal antigen testing has advantages over the urea breath test in cases where patients are unwilling or unable to undergo the breath test or have poor breath coordination (12, 13). Additionally, many hospitals have initiated genetic testing for antibiotic resistance, a crucial technical foundation for guiding personalized treatment in the fight against *H. pylori*.

This survey investigated routine *H. pylori* testing and examination methods for patients with peptic ulcers and bleeding at different hospital levels. During hospitalization, 68.79% of primary, 80.72% of secondary, and 76.01% of tertiary hospitals conducted *H. pylori* examinations. After discharge, 16.56, 17.09, and 22.45% of primary, secondary, and tertiary hospitals, respectively, performed outpatient *H. pylori* examinations. However, a relatively high proportion of primary hospitals (14.65%) did not perform *H. pylori* testing, indicating a need for improvement. Primary hospitals should strengthen their education on the etiology of peptic ulcers with bleeding and implement routine *H. pylori* testing.

Regarding diagnostic methods, the urea breath test is the most commonly used across hospitals at different levels, with a usage rate exceeding 50%. The rates of the rapid urease test, serological antibody test, and histopathological test ranged from 5 to 10%. However, in tertiary hospitals, the usage rate of serological antibody tests is only 13.04%, primarily for epidemiological investigations. Current infections can be considered in specific circumstances, such as peptic ulcer bleeding, patients with atrophic gastritis, and gastric mucosa-associated lymphoid tissue lymphoma who are positive for *H. pylori* antibodies and have not received eradication treatment (14). The development of rapid urease and pathological tests is relatively limited and requires further investigation.

In terms of treatment, the timing and protocol of *H. pylori* eradication and the selection of antibiotics were investigated in patients with peptic ulcers and bleeding at different levels of hospitals. Most hospitals at various levels chose to eradicate ulcers immediately after discharge. The percentages of immediate eradication during hospitalization and after discharge were 71.34, 83.95, and 81.74% in primary, secondary, and tertiary hospitals, respectively. After ulcer healing, the eradication rates were 17.83, 13.05, and 17.41% in the primary, secondary, and tertiary hospitals, respectively. The proportions of non-eradication of *H. pylori* in primary, secondary, and tertiary hospitals were 10.83, 3, and 0.83%, respectively, indicating that treatment in primary hospitals was highly non-standard. The *H. pylori* study group should enhance the learning of the consensus guidelines among physicians at primary hospitals.

Regarding eradication options, using bismuth-containing quadruple 14-day regimens accounted for 81.53, 89.49, and 91.42% in primary, secondary, and tertiary hospitals, respectively. This aligns with the sixth National Consensus Report on the Management of *H. pylori* Infections in China, which recommends using bismuth-containing quadruple regimens for primary and secondary eradication. The survey also revealed that the rates of choosing high-dose combination therapy for 14 days in primary, secondary, and tertiary hospitals were 7.01, 3.12, and 3.42%, respectively. A significant proportion of physicians still choose triple therapy, with an average eradication rate of 71.3% in China. Triple therapy is no longer recommended, and its continued use may be attributed to the slow updating of physician knowledge and insufficient familiarity with the latest consensus guidelines.

Regarding the choice of antibiotic regimens, the investigation found that in primary hospitals, amoxicillin + clarithromycin had the highest proportion, followed by amoxicillin + levofloxacin and amoxicillin + furazolidone. In secondary and tertiary hospitals, the most common regimens were amoxicillin + furazolidone and amoxicillin + metronidazole, respectively.

The survey results highlighted several important findings regarding *H. pylori* eradication treatments. First, physicians' preference for amoxicillin and clarithromycin regimens indicates the need for increased awareness of antibiotic resistance and the importance of conducting antibiotic sensitivity testing. The Maastricht VI/Florence Consensus recommends resistance testing before using clarithromycin-containing prescriptions (15). In China, PCR-based clarithromycin-resistance gene detection kits are available and have been used during the COVID-19 pandemic. Personalized treatments based on resistance gene testing have shown promise in improving eradication rates.

The survey also revealed high resistance rates to antibiotics commonly used for *H. pylori* eradication, such as clarithromycin, levofloxacin, and metronidazole. Furazolidone and tetracycline are relatively sensitive, but their availability is limited. Furazolidone is no longer used in many countries owing to safety concerns. Metronidazole is widely available but has a high resistance rate (16). The availability of clarithromycin and amoxicillin is high, making personalized treatment guided by clarithromycin resistance genes the preferred option.

Regarding the timing of re-examination after *H. pylori* eradication, a significant proportion of hospitals chose not to conduct follow-up examinations or scheduled them too early. Re-examination after 4 weeks of discontinuation was common, but some physicians opted for a 6-month interval to ensure accurate determination of eradication success. The lack of adherence to recommended guidelines suggests the need for further education and awareness among physicians.

In cases where *H. pylori* eradication failed, the survey explored the timing and protocol selection for re-eradication. European guidelines (17) suggest allowing *H. pylori* to regain activity after a 3–6-month interval before initiating re-eradication treatment. Hospitals have chosen various approaches, with the majority replacing eradication plans. It is crucial to consider local clinical characteristics such as drug resistance and accessibility and avoid reusing previously ineffective antibiotics.

Resistance to clarithromycin and levofloxacin is also associated with eradication failure (18). Therefore, a thorough investigation of a patient's history of antibiotic use is necessary to guide treatment decisions. Primary resistance to amoxicillin, tetracycline, and furazolidone is rare in China. The treatment duration is also an important consideration, with a 14-day course showing higher eradication rates in refractory cases.

Non-antibiotic adjunctive drugs, such as traditional Chinese medicines and probiotics, have shown the potential to reduce adverse reactions and improve patient tolerance to *H. pylori* eradication treatment (19, 20). However, further research is needed to establish their efficacy and role in improving eradication rates.

## Conclusion

It is important for physicians at all hospital levels, particularly primary hospitals, to enhance their understanding of *H. pylori* infection, eradication, follow-up, and re-eradication. The Maastricht VI/Florence and Chinese National Consensus reports provide valuable guidelines for *H. pylori* management and should be incorporated into clinical practice.

It should be noted that this survey was conducted after the publication of the Maastricht VI/Florence Consensus and the Chinese National Consensus Report, indicating the need for continued education and implementation of the latest guidelines. This study had some limitations, including the relatively small proportion of primary hospitals, which should be addressed in future research.

## Data Availability

The original contributions presented in the study are included in the article/supplementary material, further inquiries can be directed to the corresponding author.
